# The Effect of Human Platelet-Rich Plasma on Adipose-Derived Stem Cell Proliferation and Osteogenic Differentiation

**DOI:** 10.6091/ibj.1301.2014

**Published:** 2014-07

**Authors:** Sima Tavakolinejad, Mohsen Khosravi, Baratali Mashkani, Alireza Ebrahimzadeh Bideskan, Nasser Sanjar Mossavi, Seyyed Mohammad Reza Parizadeh, Daryoush Hamidi Alamdari

**Affiliations:** 1 *Dept. of Anatomy and Cell Biology, School of Medicine, Mashhad University of Medical Sciences, Mashhad, Iran;*; 2 *Dept. of Clinical Biochemistry, Mashhad University of Medical Sciences, Mashhad, Iran; *; 3 *Dept. of Surgery, School of Medicine, Islamic Azad University, Mashhad Branch, Iran; *; 4 *Stem cell and Regenerative Medicine Research Group, Biochemistry and Nutrition Research Center, Dept. of Clinical Biochemistry, School of Medicine, Mashhad University of Medical Sciences, Mashhad, Iran*

**Keywords:** Platelet-Rich Plasma, Adipose tissue, Stem Cells, Cell differentiation, Cell proliferation

## Abstract

**Background**: The cultured mesenchymal stem cells (MSC) have been used in many clinical trials; however, there are still some concerns about the cultural conditions. One concern is related to the use of FBS as a widely used xenogeneic supplement in the culture system. Human platelet-rich plasma (hPRP) is a candidate replacement for FBS. In this study, the effect of hPRP on MSC proliferation and osteogenic differentiation has been evaluated. **Methods**: Human adipose-derived stem cells (hADSC) were expanded. Cells from the third passage were characterized by flow cytometric analysis and used for *in vitro* experiments. Resazurin and alizarin red stains were used for cell proliferation and osteogenic differentiation assays, respectively. **Results**: Treatment with hPRP resulted in a statistically significant increase in cell proliferation compare to the negative control group (*P*<0.001). Cell proliferation in the 15% hPRP group was also significantly higher than that in the 10% hPRP group (*P*<0.05). Additionally, it caused less osteogenic differentiation of the hADSC compared to the FBS (*P*<0.001), but in comparison to negative control, it caused acceptable mineralization (*P*<0.001). **Conclusion**: These findings indicate that hPRP not only improves the proliferation but also it can be a suitable substitution in osteogenic differentiation for clinical purposes. However, the clinical application value of hPRP still needs more investigation.

## INTRODUCTION

Following promising results of mesenchymal stem cells (MSC) in experimental studies and clinical trials, these cells have been recently presented as a candidate for cell-based therapy in solid organ transplantation and repair of damaged tissues such as bone, cartilage, muscle, ligament, tendon, and adipose [1]. Because of their immunosuppressory and engraftment-promoting properties, MSC have been used routinely in the clinical settings both to facilitate hematopoietic recovery and to treat steroid-resistant acute graft-versus-host disease [2]. Compared to other types of stem cells, MSC are considered as a useful cell source in tissue engineering owing to their high capacity for proliferation and differentiation [1, 3]. Bone marrow-derived mesenchymal stem cells have been studied extensively. In addition, human adipose-derived stem cells (hADSC) have recently attracted more attention, because they can be acquired easily in large quantities with minimal morbidity. hADSC possess greater potential for proliferation in comparison to bone marrow-derived mesenchymal stem cells [4, 5].

Due to some concerns about the ingredients in the culture media, many attempts have been made to optimize the culture condition for clinical purposes. One such concern is related to the use of FBS, which introduces a wide range of foreign proteins into the cultural system and more importantly has the potential risk of transmitting infectious agents such as prions. Therefore, many research has been carried out to find a suitable alternative for FBS [6]. The human platelet-rich plasma (hPRP) supplement might be a good candidate to replace FBS, and autologous hPRP can be easily produced at low cost. Its heterologous supplies also induce a poor immune response. *In vitro* studies have confirmed that hPRP improves proliferation of a variety of cell types such as MSC [6, 7]. Platelets contain many cytokines, including platelet-derived growth factor, transforming growth factor beta, insulin-like growth factor-1, and vascular endothelial growth factor in their α-granules. These cytokines promote wound healing and repair damaged bones [8]. As the concentration of platelets rises in hPRP, the amount of cytokine increases accordingly [6]. Meanwhile, autologous hPRP has a significant advantage in regenerative medicine. In this study, the effects of various concentrations of buffered hPRP on hADSC proliferation and osteogenic differentiation have been determined. 

## MATERIALS AND METHODS

***Tissue collection.*** Human adipose tissue was taken from donors (n = 6) undergoing liposuction surgery, Razavi Hospital, Mashhad, Iran. All donors were healthy Persian females, ages ranging from 30 to 40 years. This procedure was approved by the Institutional Ethics Committee of Mashhad University of Medical Sciences (Mashhad, Iran), and an informed consent was obtained from all donors. To avoid bias, each step was performed by one person, and all devices were calibrated.

***Cell isolation and culture. ***hADSC were extracted from the human adipose tissue. Briefly, the cells were washed with PBS and digested by 0.01% collagenase Type-1 at 37^o^C for 1.5 hours. The cells were sedimented at 600 ×g at 25^o^C for 15 min. Tris lysing buffer was used to remove red blood cells. Finally, hADSC were cultured in αMEM (Gibco, Invitrogen Carlsbad, CA) supplemented with 10% (v/v) FBS and 1% penicillin/ streptomycin (Sigma, St. Louis, MO). The culture medium was replaced every 3 days. The cells were detached using trypsin-EDTA (Gibco, Invitrogen Carlsbad, CA) at 70% confluency and sub-cultured to the new flasks. The cells from third passage were used for characterization by flow cytometry and *in vitro* experiments [6, 9-12]. 

***Flow cytometric characterization of human adipose -derived stem cells. ***hADSC showed to be positive for several CD markers, including CD9, CD29, CD49, CD54, CD105, CD166, CD44, CD71, CD10, CD13, CD73, CD90, CD59, CD146, and CD55 and negative for some others, such as CD18, CD50, CD11b, CD56, CD62, CD45, CD104, CD16, CD14, and CD31, which can be defined for phenotypic characterization. Also in other studies, the differentiation potential into chondroblast, adipoblast and osteoblast have been examined [11, 13]. However, in this study, hADSC were assessed for the expression of CD29 and CD90. Briefly, the hADSC were harvested and stained with FITC-conjugated mouse anti-human CD29 and phycoerythrin-conjugated mouse anti-human CD90 at room temperature for 15 min. Two other groups of cells were stained with FITC or phycoerythrin-labeled mouse anti-human IgG (all from Serotec, Oxford, UK) as negative controls. Finally, the stained cells were used for flowcytometry (BD Biosciences, San Jose, CA, USA), followed by data analysis using WinMDI 2.9 software.

**Fig. 1:**
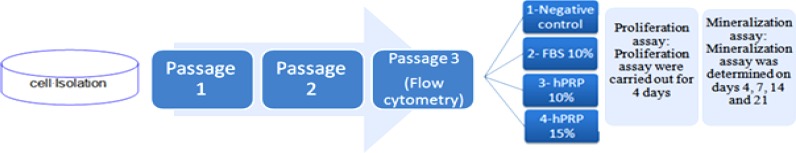
The flow chart of the study: hADSC were isolated from adipose tissue. The cells were sub-cultured until the third passage and characterized by flow cytometry before being divided into four groups. Then cell proliferation and differentiation assays were carried out. The culture media for all groups were αMEM and 1% antibiotics. Negative group contained neither FBS nor hPRP, and positive group contained 10% FBS. Groups 3 and 4 contained 10% and 15% hPRP, respectively. hPRP, human platelet-rich plasma, FBS, fetal bovine serum

***Experimental groups. ***As described in Figure 1, there were four experimental groups: 1) negative control (no supplement was added), 2) 10% FBS (positive control), 3) 10% hRPR, and 4) 15% hPRP. All other culture conditions were the same among the groups. We had evaluated the proliferation and osteogenesis between these groups using both normal and osteogenic culture media.

***Preparation of activated hPRP. ***Human blood (450 ml) was collected in triple blood bag. The PRP preparation procedure was consisted of two centrifugation steps. After the first centrifugation, the platelet-rich plasma was transferred to other bag. In the second centrifugation step, the platelets were precipitated, and the plasma was removed, then the platelets were resuspended in 50 ml plasma. 

Afterward, for hPRP activation, the platelet and plasma mixture was frozen and thawed 6 times, then centrifuged at 3,000 ×g for 5 min in order to remove particles. The activated hPRP (hPRP-released growth factors in supernatant) was stored at -70^o^C until use. Before using sodium, bicarbonate (Gibco, Invitrogen Carlsbad, CA) was added to neutralize the pH [6]. 

***Cell proliferation assay. ***Resazurin assay was used to measure cell proliferation after the third passage. In this assay, the cells were seeded in 96-well plates in triplicate at a density of 5 × 10³ cell per well in 200 µl culture media supplemented with either FBS or hPRP as described for groups 1-4. The plates were incubated in 5% CO_2_ at 37°C for four days. A volume of 20 μl resazurin reagent was added daily to appropriate wells and then incubated for 5 hours. Finally, the fluorescent intensity (530Ex/590Em) was measured by a plate reader (Perkins Elmer, Finland) [14-18].

***Mineralization assay. ***Mineralization was determined using alizarin red stain solution on days 4, 7, 14, and 21. After the third passage, the cells were seeded in a 24-well plate in culture media containing either FBS or hPRP as described earlier for groups 1-4. To stimulate osteogenesis in all groups, 100 nM 

dexamethasone, 5 µM ascorbic acid, 0.5 mM β-glycerophosphate, and antibiotics (100 U/ml penicillin and 100 mg/ml streptomycin) were also added to the culture media. For evaluation of mineralization, cells were washed with PBS and fixed with 4% paraformaldehyde for 15 minutes. After rinsing with excess distilled water, alizarin red solution was added (1 ml/well) and incubated at room temperature for 20 minutes. After wash, 400 µl of 10% acetic acid was added into each well to extract the dye, and the absorbance was measured at 405 nm by a plate reader (Perkins Elmer, Finland) [15, 19].

***Statistical analysis. ***The data analysis was performed using SPSS 11.5, which included one-way analysis of variance (ANOVA) and post-hoc Tukey test. If variances were not homogeneous, the Kruskal-Wallis test was used, followed by the Nemenyi test. Statistical significance was determined based on* P values* less than 0.05.

## RESULTS

**Fig. 2:**
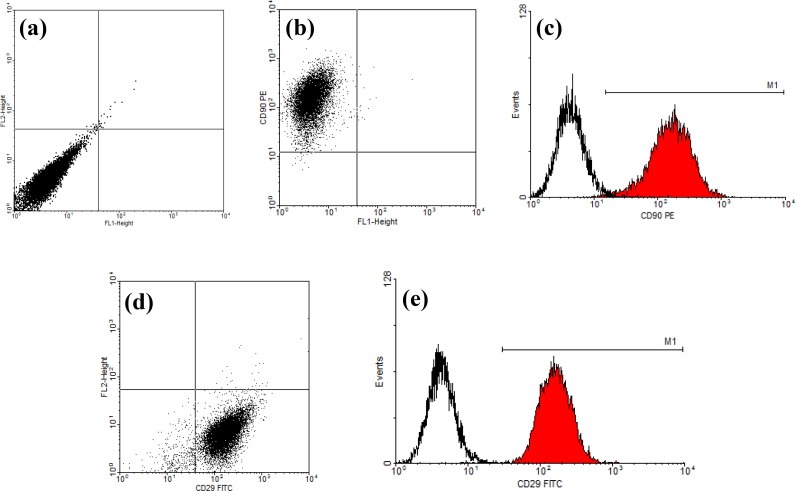
Flow cytometry of hADSC after the third passage. **(a)** Dot plot of unstained cells; (**b** and **c**) dot plot and histogram of CD90 phycoerythrin-conjugated stained cells, respectively (99.61% of the cells was positive); (**d** and **e**) Dot plot and histogram of CD29 FITC-conjugated stained cells, respectively (95.25% of the cells was positive for CD29). Negative controls are shown in histograms (the first pick in c and e).

***Flow cytometry analysis. ***Flow cytometric analysis of the passage 3 of hADSC confirmed the expression of both CD29 and CD90 on the cell surface (Fig. 2).

**Fig. 3:**
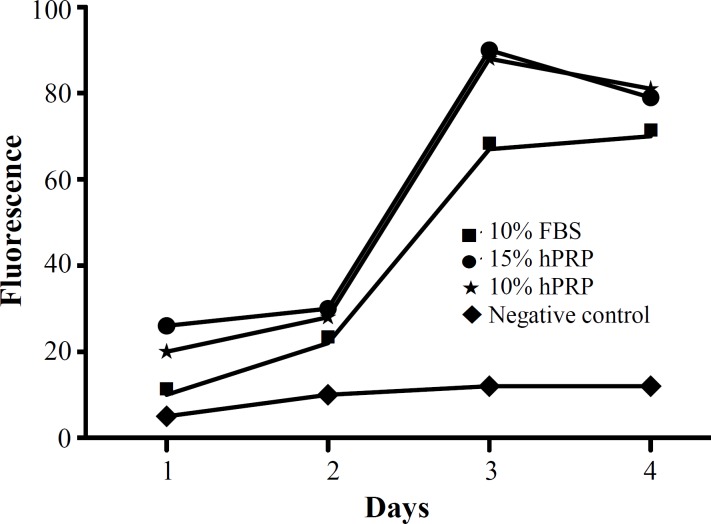
Cell Proliferation rate in different experimental groups mentioned in the box (fluorescence scale is multiplied by 10^4^) hPRP, human platelet-rich plasma, FBS, fetal bovine serum

***Proliferation assay. ***The florescent intensity indicating the number of live cells in each group for four consecutive days is represented in Figure 3. In the negative control group, the number of live cells has not changed during the course of this study. In comparison to 10% FBS, 10% and 15% hPRP showed a significant increase in cell proliferation at the third day.

**Fig. 4:**
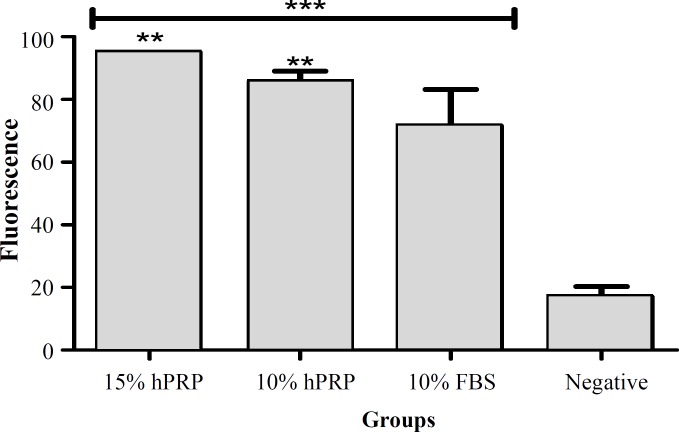
The viability of human adipose-derived stem cells on day 3 (**P<0.01, ***P<0.001) (fluorescence scale is multiplied by 10^4^). hPRP, human platelet-rich plasma, FBS, fetal bovine serum

Figure 4 represents the number of live cells on day 3 in each group. The number of live cells in groups supplemented with either FBS of hPRP was significantly higher than the negative control (*P*<0.001). The most potent supplement for promoting hADSC proliferation was 15% hPRP, followed by 10% hPRP and 10% FBS.

**Fig. 5:**
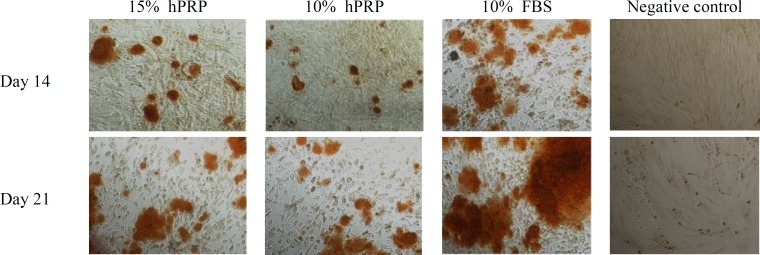
Osteogenic differentiation performed by alizarin red staining in different experimental groups as described in Figure 1 at 14 and 21 days. hPRP, human platelet-rich plasma, FBS, fetal bovine serum

**Fig. 6:**
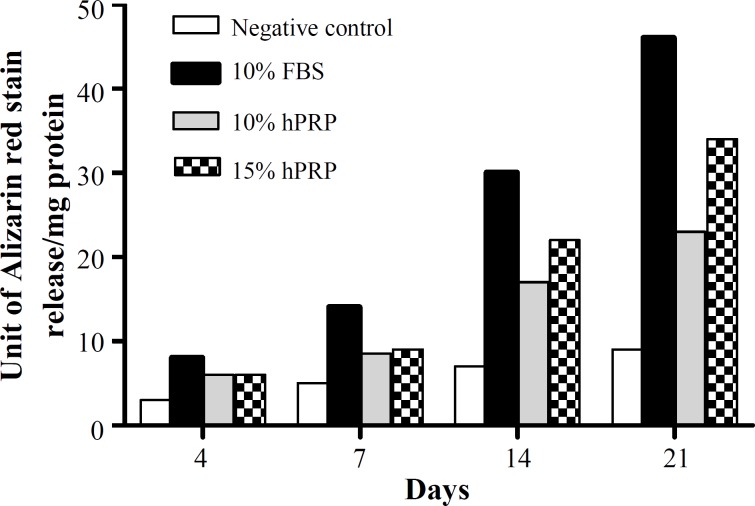
Quantitative mineralization assay in different experimental groups as described in Figure 1. FBS, fetal bovine serum, hPRP, human platelet-rich plasma

***Mineralization assay. ***Mineralization of the hADSC was occurred when the osteogenesis induction was performed in the media supplemented with either FBS or hPRP (Fig. 5). The quantitative determination of calcium deposits on days 4, 7, 14, and 21 is shown in Figure 6. The most potent mineralization-inducing agent was 10% FBS, followed by 15 and 10% hPRP. On days 14 and 21, there was a significant increase in the mineralization with the cultural media containing hPRP compared with the negative control (*P*<0.001). At 14 and 21 days, 15% hPRP group significantly had higher mineralization than 10% hPRP group (*P*<0.05). However, the mineralization rate on days 14 or 21 with both 10 and 15% hPRP was significantly lower than that with 10% FBS (positive control) (*P*<0.001) (Table 1).

## DISCUSSION

This study shows that FBS can be replaced by hPRP, and the treating cells with 10% and 15% hPRP induce high levels of proliferation and less osteogenic differentiation compared to FBS. Three criteria needed to be considered for clinical applications of stem cells. First, these cells must be sufficiently expanded for clinical transplantation. Second, immune rejection should be avoided after transplantation, and third non-xenogeneic supplement should be used for *ex vivo* proliferation and differentiation of MSC [9].

FBS, a xenogeneic supplement, can be used for proliferation and osteogenic differentiation of MSC that gives acceptable results [15]. However, due to some concerns about immunogenicity and also transmission of infectious agent including prions, FBS is not the best choice for clinical applications. Nowadays, many studies have been focused on the use of growth factors released from hPRP as a powerful substitute for FBS in various types of cell cultures, such as MSC, fibroblasts, and osteoblasts [8, 20-23]. hPRP can be easily prepared from autologous peripheral blood with a large quantity and minimal donor site morbidity [8]. However, there are still some conflictive results in different studies on the efficacy of hPRP [8, 21], which may result from different preparation technique for hPRP. 

Two procedures have been used to activate hPRP. In some studies [23, 24], calcium and thrombin were used, and in another study [25], several freezing and thawing methods were performed to activate hPRP. The elimination of thrombin from hPRP preparation is very important, because bovine thrombin has been shown to have significant immunological side effects [6, 26]. In this study, several freezing and thawing methods were used for activation of platelets. In a prior investigation, it was noted that acidic hPRP (pH 6.8-7.1) causes altered action of cytokines [27]. hPRP with alkaline pH also releases additional components with stimulating effects on cell proliferation [28]. Considering these reports, the hPRP was buffered to physiologic pH (7.4) in this study.

There are many studies which had assessed the effect of hPRP on the viability and proliferation of cells, and the results showed stimulation of cell proliferation by hPRP [7, 24, 29, 30]. It was demonstrated that hPRP enhances MSC proliferation without compromising differentiation capacity and immunephenotype [7]. Our study also indicates that hPRP stimulates the proliferation of hADSC compared to the positive control (10% FBS), which is consistent with the previous reports [7, 24, 29, 30]. The highest rate of proliferation was achieved using 15% hPRP, which was almost equal to the rate obtained with 10% FBS.

Mineralization is a reliable marker for osteogenic differentiation and bone formation [31]. In our study, the highest mineralization rate was in 10% FBS, followed by 15 and 10% hPRP. These results are consistent with the results reported previously from other research [7, 15, 30, 32] that indicated that the effect of hPRP on the mineralization of cells was significantly lower than that of FBS. The study of Li *et al.* [30] showed that autogeneic hPRP could induce osteogenic differentiation. We also indicated that the osteogenic differentiation of cells cultured in allogeneic hPRP contained media, but the rate of differentiation was dose dependent. However, according to our findings, the most promising osteogenic differentiation was related to FBS containing cell culture media. 

Moreover, it has been reported that PRP-derived from human umbilical cord blood could promote proliferation and osteogenic differentiation of MSC cells [9], which is inconsistent with our findings. In addition, some other studies found PRP promoting for osteogenic differentiation, but it might be due to application of synergic factors [33, 34]. 

Our results show that FBS can be replaced by hPRP to induce cell proliferation. The hPRP effects on cell proliferation were dose dependent with 15% hPRP having the highest proliferation rate. Although FBS seems to be more effective than hPRP on osteogenic differentiation of hADSC, less osteogenic differen-tiation effect of hPRP may be a positive indication for using these cells. In other applications, the cultured MSC should be more naïve and look like the uncultured primary MSC. Replacements of FBS with PRP eliminate the risks presented by the use of xenogene media supplements. Therefore, hPRP may be considered in place of FBS in regenerative medicine for clinical purposes.
